# *Chryseobacterium aquifrigidense* FANN1 Produced Detergent-Stable Metallokeratinase and Amino Acids Through the Abasement of Chicken Feathers

**DOI:** 10.3389/fbioe.2021.720176

**Published:** 2021-08-06

**Authors:** Amahle Bokveld, Nonso E. Nnolim, Uchechukwu U. Nwodo

**Affiliations:** ^1^ SAMRC Microbial Water Quality Monitoring Centre, University of Fort Hare, Alice, South Africa; ^2^ Applied and Environmental Microbiology Research Group (AEMREG), Department of Biochemistry and Microbiology, University of Fort Hare, Alice, South Africa

**Keywords:** amino acids, bio-additive, bioeconomy, chicken feathers, keratinase, protein hydrolysate

## Abstract

Microbial keratinases’ versatility in the beneficiation of keratinous waste biomass into high-value products prompts their application in diverse spheres hence, advancing green technology and the bioeconomy. Consequently, a feather-degrading *Chryseobacterium aquifrigidense* FANN1 (NCBI: MW169027) was used to produce keratinase, and its biochemical properties were determined. The optimization of physicochemical parameters and analysis of the free amino acid constituents of the feather hydrolysate were also carried out. FANN1 showed a maximum keratinase yield of 1,664.55 ± 42.43 U/mL after 72 h, at optimal process conditions that included initial medium pH, incubation temperature, inoculum size, and chicken feather concentration of 8, 30°C, 4% (v/v), and 15 (g/L), respectively. Analysis of degradation product showed 50.32% and 23.25% as the protein value and total free amino acids, respectively, with a relatively high abundance of arginine (2.25%) and serine (2.03%). FANN1 keratinase was optimally active at pH 8.0 and relatively moderate to high temperature (40–50°C). EDTA and 1,10-phenanthroline inhibited the keratinase activity, and that suggests a metallo-keratinase. The enzyme showed remarkable stability in the presence of chemical agents, with residual activity 141 ± 10.38%, 98 ± 0.43%, 111 ± 1.73%, 124 ± 0.87%, 104 ± 3.89%, 107 ± 7.79%, and 112 ± 0.86% against DTT, H_2_O_2_, DMSO, acetonitrile, triton X-100, tween-80, and SDS, respectively. The residual activity of FANN1 keratinase was enhanced by Sunlight (129%), Ariel (116%), MAQ (151%), and Surf (143%) compared to the control after 60 min preincubation. Likewise, the enzyme was remarkably stable in the presence Fe^3+^ (120 ± 5.06%), Ca^2+^ (100 ± 10.33%), Na^+^ (122 ± 2.95%), Al^3+^ (106 ± 10.33%); while Co^2+^ (68 ± 8.22%) and Fe^2+^ (51 ± 8.43%) elicited the most repressive effect on keratinase activity. The findings suggest that *C. aquifrigidense* FANN1 is a potential candidate for keratinous wastes bio-recycling, and the associated keratinase has a good prospect for application in detergent formulation.

## Introduction

In recent years, investment increase in the agro-industrial sector is evident for food security sustenance for the teeming population. Consequently, the vast tons of agro-wastes generated from this economic sector have been a global concern due to their associated environmental challenges (Falade, 2021). Non-food agro-waste residues with sturdy characteristics have become a burden to society and the environment. Hence, the need for sustainable beneficiation and management approach for these renewable resources. The poultry industry is a significant player as per contribution to the substantial agro-waste biomass generated. Most of the wastes from the poultry processing farms are keratinous. The abundance of keratin in chicken feathers impairs the natural degradability of the biomass in the environment.

The chicken feather beneficiation process consequent to the rich keratin content includes conversion into feather meals for animal feed supplementation (Akram et al., 2021). However, the thermo-chemical-mechanical approach for valorizing chicken feathers into feedstock supplements compromises nutrients’ integrity and involves high energy input (Abdel-Fattah et al., 2018). The keratinous agro-residues valorization approach involving the application of keratinolytic microbes is considered sustainable and environmentally friendly (Nnolim et al., 2020a). Amino acid-rich protein hydrolysates from the microbial dismemberment of keratinous residues serve as cheap protein supplements to formulate livestock feeds (Adetunji and Adejumo, 2018). They could also be utilized in the pharmaceutical sector for biosynthesizing essential vitamins using a suitable industrial workhorse (Hassan et al., 2020). Keratinolytic protease has been used in feeds to improve the nutrients’ bioaccessibility and bioassimilability (Wang et al., 2006; Adetunji and Adejumo, 2018). The versatility of microbial keratinase accounts for the array of applications of the enzyme in advancing green technological processes in the bio-economy (Nnolim et al., 2020a).

Keratinases production has been reported in autochthonous strains of *Bacillus* sp., *Streptomyces* sp., *Arthrobacter* sp., and *Chryseobacterium* sp. from diverse ecological niches (Hassan et al., 2020), and these microbes and their products assessed for suitability in non-polluting bioprocesses, including animal skin dehairing, keratin hydrolysis, feed production, wastewater treatment, green nanoparticle synthesis, and detergent formulation (Gupta et al., 2015; Reddy et al., 2017; Hamiche et al., 2019; Zhang et al., 2019). Crude protein hydrolysates from biodegradation of keratinous wastes have been under extensive evaluation as substitutes for inorganic fertilizer to grow cash crops due to their high nitrogen content and other vital micronutrients that enhance plant growth and development (Gurav et al., 2020). Soil amendment with this bioresource promotes soil fertility while ensuring sustainable agroecology (Gurav and Jadhav, 2013a). Keratinolytic proteases have shown high tolerance to surfactants and other laundry detergents than the classical proteases used in commercial detergent formulation (Benkiar et al., 2013; Paul et al., 2016). Detergent formulation with keratinolytic enzymes may improve, significantly, washing performance as keratinase facilitates hydrolysis of hard and soft proteinaceous stains with mild effect on the fabrics (Paul et al., 2014). Keratinase has the potentials to undoubtedly dominate the protease market because of its robustness and the anticipated cost-effective production processes following agro-industrial waste beneficiation. Strain peculiarity informs keratinase properties; hence, the need to explore the environment for microbes with the propensity for novel and high titre keratinases.

The dexterity for which keratinous biomass was beneficiated through dismemberment into high-value products by strains of *Chryseobacterium* spp. has been reported (Kang et al., 2018; Kang et al., 2021). Consequently, *Chryseobacterium aquifrigidense* FANN1 isolated from the poultry dumpsite was assessed for keratinase production. In addition, keratinase activity optima and catalytic properties and stability profile in chemical agents and laundry detergents were evaluated.

## Materials and Methods

### Bacterial Isolate

The chicken feather-degrading bacterium used for the study was isolated from a soil sample collected from a poultry dumpsite (Unpublished). The isolate identity was confirmed as *Chryseobacterium aquifrigidense* FANN1 (accession number MW169027.1) based on 16S rRNA gene sequencing with a set of universal oligonucleotides - 27f (5′-AGAGTTTGATCMTGGCTCAG-3′) and 1492r (5′-CGG​TTA​CCT​TGT​TAC​GAC​TT-3′) (Turner et al., 1999) and phylogenetic analysis.

### Keratinase Production

Keratinolytic protease production was carried out using a basal salt medium (BSM), containing 0.3 g K_2_HPO_4_, 0.4 g KH_2_PO_4_, 0.2 g MgCl_2_, 0.22 g CaCl_2_ in 1 L of distilled water. BSM (50 ml) was dispensed in 250 ml Erlenmeyer flasks and was supplemented with 15 g/L of pulverized chicken feathers (PCF). The keratinous substrate–PCF was prepared as described previously (Nnolim et al., 2020b). The flasks were sterilized, and after that, the initial medium pH was aseptically adjusted to 6. The flasks were inoculated with 4% (v/v) of the standardized bacterial suspension (optical density of 0.1 at 600 nm) and incubated at 30°C in a rotary shaker (150 rpm) for 72 h. After incubation, the crude keratinase was extracted by centrifuging the fermentation broth at 15,000 rpm for 10 min and 4°C. The crude extract was used for further characterization studies.

### Keratinase Activity Assay

The keratinase activity assay followed the method described by Nnolim et al. (2020b). Briefly, the reaction mixture contained 0.5 ml of a freshly prepared crude enzyme and 0.5 ml of 10 g/L of keratin azure in Tris-HCl (pH 8; 0.1 M). The reaction mixture was incubated at 50°C under a constant shaking condition (220 rpm). After 1 h of incubation, the reaction was stopped by placing the reaction mixture on an ice bath for 10 min. Next, the mixture was centrifuged at 15,000 rpm for 10 min. Finally, an aliquot of the supernatant was used to determine the absorbance at 595 nm using an SYNERGYMx 96 well microplate reader (BioTek, United States).

### Protein and Thiol Contents Quantitation

The total protein content of the crude extract was quantified following Bradford protocols using bovine serum albumin as the standard protein (Bradford, 1976). In addition, the thiol group liberated in the cell-free broth during feather keratin dismemberment was quantified with 5,5′-dithio-bis-(2-nitrobenzoic acid), as reported by Ellman (1959).

### Optimization of Significant Process Variables

The significant process variables were studied using one variable at a time (OVAT) method. The initial medium pH and incubation temperature were varied from 3.0–9.0 and 25–50°C at intervals of 1 unit and 5°C, respectively. The effect of inoculum size on keratinase production was determined using the standardized bacterial suspension at a range of 1–7% (v/v; at intervals of 1%), while the chicken feather concentration was varied from 5 g/L to 35 g/L (at intervals of 5 g) to establish the best concentration that would enhance extracellular keratinase production.

After establishing the optimal process variables, the time course profile of keratinolytic activity of *C. aquifrigidense* FANN1 was studied. The fermentation was carried out in 500 ml Erlenmeyer flasks with a working volume of 100 ml BSM at the optimal process variables. Aliquots were aseptically withdrawn at intervals (24 h) to determine various parameters tested.

### Determination of Free Amino Acids Content of the Feather Hydrolysate

The feather hydrolysate extracted from the culture broth by centrifugation was freeze-dried in a vacuum concentrator (Martin Christ Gefriertrocknungsanlagen GmbH, Germany). The lyophilized powder was used to determine the free amino acid composition by pre-column derivatization, high-performance liquid chromatography (HPLC) separation, and quantification as previously described (DeVries et al., 1980; Einarsson et al., 1983; Gehrke et al., 1985).

### Biochemical Properties Determination

#### Effect of pH on the Activity and Stability of Keratinase

Keratinase activity was determined over a pH range of 5.0–11.0 with the following buffer solutions (100 mM): sodium citrate; pH 5.0, potassium phosphate; pH 6.0–7.0, Tris-HCl; pH 8.0–9.0 and glycine-NaOH; pH 10.0–11.0. After that, the enzyme’s pH stability was evaluated by pre-incubating the enzyme with buffer solutions with pH 8.0 and pH 9.0 at 30°C for 4 h. Aliquots were withdrawn at 30 min intervals, and the residual activity was determined under the standard assay conditions. The enzyme activity determined without preincubation served as the control.

#### Effect of Temperature on the Activity and Stability of Keratinase

The effect of temperature on keratinase activity was determined by carrying out standard keratinase activity assays at different temperatures ranging from 30°C to 80°C. In addition, the thermostability of the keratinolytic enzyme was evaluated by preheating the enzyme solution at 40°C and 50°C for 2 h. Then, aliquots were withdrawn at intervals of 30 min to determine the residual enzyme activity under the standard assay conditions.

#### Effect of Chemical Agents on Keratinase Stability

The enzyme solution was pre-incubated with protease inhibitors, including phenylmethylsulfonyl fluoride (PMSF), ethylenediaminetetraacetic acid (EDTA), 1,10-phenanthroline at 5 mM. Similarly, 5 mM of dithiothreitol (DTT), sodium dodecyl sulfate (SDS), and 1% (v/v) of hydrogen peroxide, acetonitrile, dimethyl sulfoxide (DMSO), triton X-100, tween-80 were preincubated with an admixture of enzyme solution for 1 h at 30°C. Post-residual activity determination was carried out under the standard assay protocols.

#### Effect of Metal Ions on the Catalytic Efficiency of Keratinase

A group of metal ions used to evaluate the enzyme stability included Mg^2+^, Co^2+^, Fe^3+^, K^+^, Ca^2+^, Mn^2+^, Fe^2+^, Cu^2+^, Zn^2+^, Ba^2+^, and Al^3+^. The chloride salts of these metal ions were pre-incubated with the keratinolytic protease at 5 mM for 1 h. After incubation, the residual activity was determined under the standard assay conditions. The enzyme solution preincubated with distilled water served as the control.

#### Effect of Solid Laundry Detergents on Keratinase Stability

The effect of selected laundry detergents on keratinolytic protease stability was studied using the following commercial laundry detergent, including Sunlight, Omo, Ariel, MAQ, Surf, Sky, and Pro wash. The detergents were solubilized in tap water and mixed with the enzyme solution with a final concentration of 0.7% (w/v). The enzyme contents of the detergents were inactivated by heating at 100°C for 30 min prior to mixing with keratinolytic protease. The detergent-enzyme solutions were pre-incubated for 1 h at 30°C, and aliquots were withdrawn at 30 min intervals; then, residual activity was determined under the standard assay conditions. The enzyme solution preincubated with tap water along with the test solutions served as the control experiment.

### Analysis of the Statistics

The analysis of datasets generated from triplicate experiments was conducted in the Statistical Package for the Social Science (SPSS) version 23, and the results were presented as the mean and standard deviation. The statistical difference was compared at *p* < 0.05.

## Results

### Optimization of Fermentation Conditions

**FIGURE 1:**
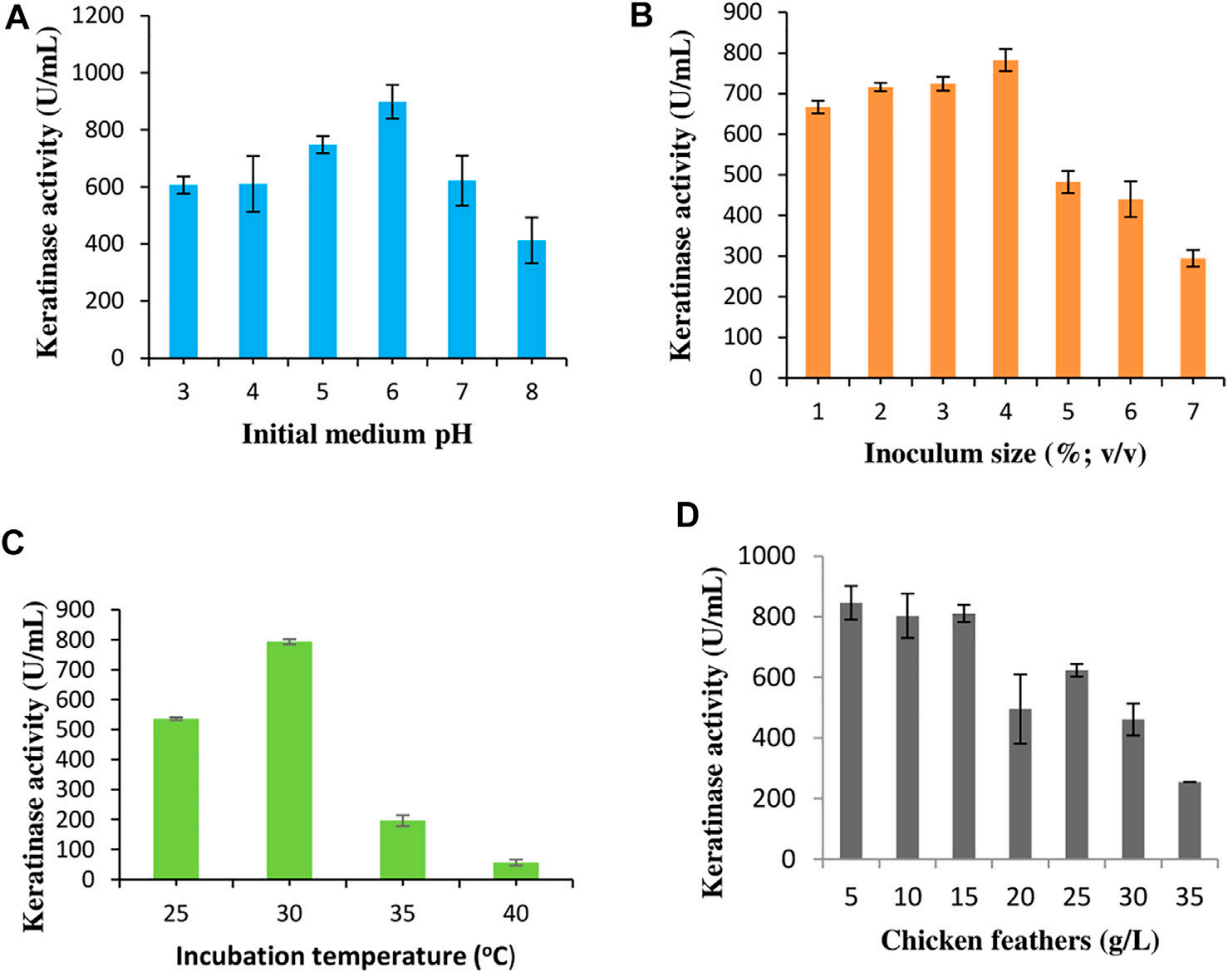
The effect of process conditions **(A)** initial medium pH, **(B)** inoculum size, **(C)** incubation temperature, and **(D)** chicken feather concentration on keratinase production by *C. aquifrigidense* FANN1.

Four significant process variables were optimized to enhance extracellular keratinase production by *C. aquifrigidense* FANN1 in BSM. The pH optimization study showed that keratinase was expressed by FANN1 at pH 3.0 (606.36 ± 29.56 U/mL), reaching optimum at pH 6.0, with the keratinase activity of 898.18 ± 59.14 U/mL (Figure 1A). Beyond pH 6.0, the extracellular keratinase secretion declined considerably. The inoculum size evaluation indicated that keratinase activity increased in a stepwise manner from 667.27 ± 15.42 U/mL, 716.36 ± 10.28 U/mL, 724.55 ± 16.71 U/mL to 782.72 ± 26.99 U/mL following the increase in bacterial cell concentrations from 1 to 4% (v/v), respectively (Figure 1B). An increment above 4% resulted in a decrease in keratinase activity. The influence of incubation temperature was investigated, and the results showed that the keratinase production was maximum (793.64 ± 8.99 U/mL) at 30°C (Figure 1C). Above 30°C, the extracellular keratinase production by *C. aquifrigidense* FANN1 was remarkably repressed. Chicken feather concentrations used for the fermentation process were varied, and the results indicated that extracellular keratinase production by FANN1 was relatively constant between 5 g/L and 15 g/L of chicken feathers (Figure 1D). Further increase in chicken feathers concentration caused a decline in the extracellular keratinase titre, with the least enzyme activity (254.54 ± 0 U/mL) obtained at the highest feather concentration (35 g/L) evaluated.

### Time Course Study of Keratinolytic Activity of *C. aquifrigidense* FANN1

**FIGURE 2:**
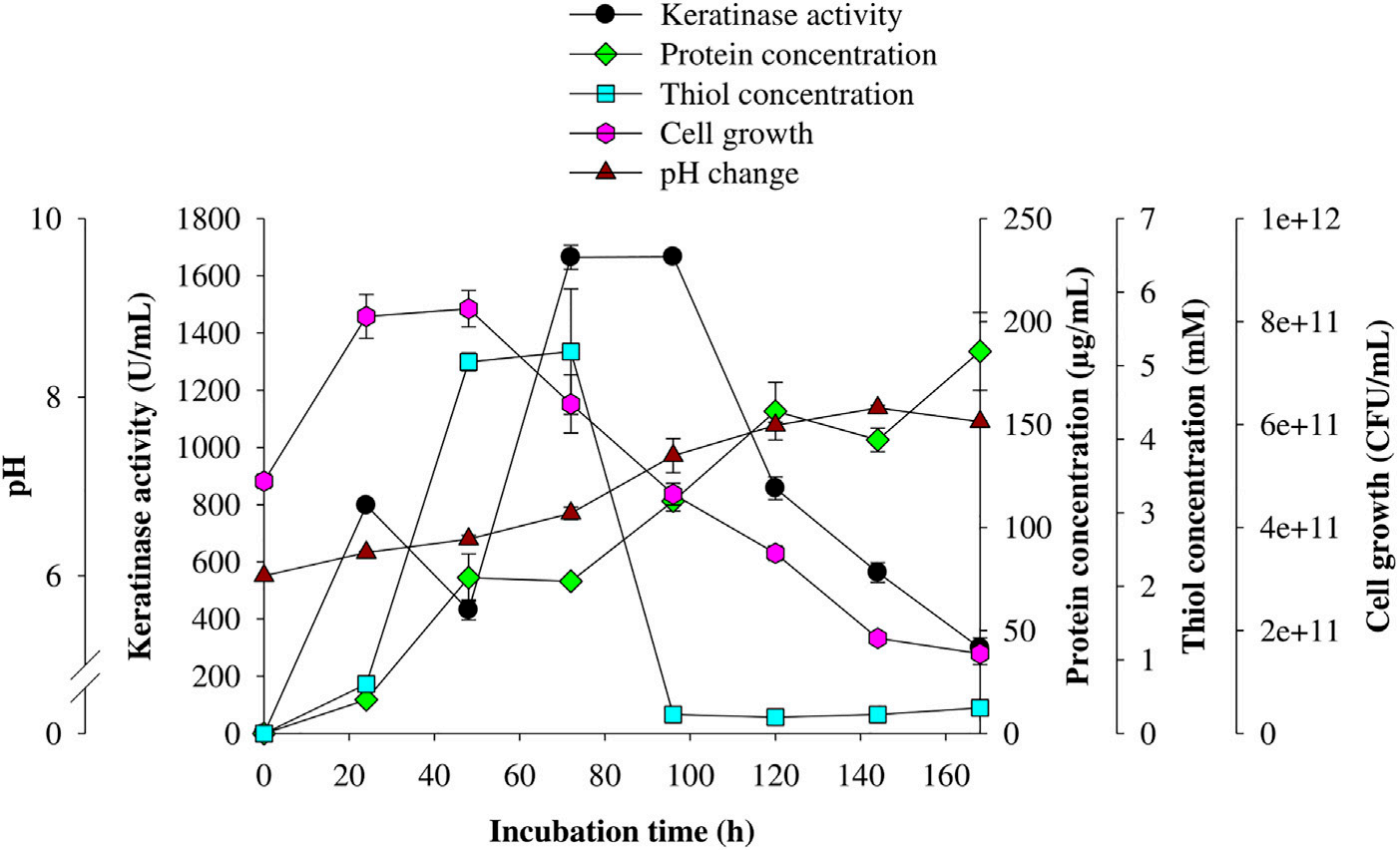
Time course profile of keratinolytic activity of *C. aquifrigidense* FANN1. The fermentation was carried out at optimal process conditions, and the experiment was performed in triplicate.

The kinetic study of keratinase production by *C. aquifrigidense* FANN1 showed that after 24 h of fermentation, the extracellular keratinase activity reached 797.27 ± 11.57 U/mL; then decreased to 431.82 ± 34.72 U/mL 48 h post-fermentation (Figure 2). Subsequently, enzyme production reached the maximum at 72 h of fermentation, with the keratinase activity of 1,664.55 ± 42.43 U/mL. After 96 h, the enzyme concentration decreased consistently. The viable cell biomass showed maximum concentration at 24 h and decreased after 48 h of incubation (Figure 2). Furthermore, thiol groups quantified during the time course study showed the peak concentrations of 5.05 ± 0.12 mM and 5.19 ± 0.85 mM at 48 and 72 h, respectively; and delined afterward (Figure 2). In addition, the total protein content of the medium increased as the fermentation progressed, reaching the maximum concentration (185 ± 18.91 µg/ml) at 168 h (Figure 2). Similarly, the pH of the production medium consistently increased over the incubation period, with the highest value of 7.88 ± 0.03 after 144 h (Figure 2).

### Amino Acid Profile of the Feather Hydrolysate

The analysis of the hydrolysate (with protein value of 50.32%) generated from chicken feathers degradation by keratinolytic *C. aquifrigidense* FANN1 showed various amino acids (total concentration of 23.25%), with a relatively higher concentration of arginine (2.25%) and serine (2.03%). Other amino acids in considerable concentration include glutamic acid (1.65%), glycine (1.93%), proline (1.69%), valine (1.67%), and leucine (1.71%), as shown in Table 1. However, methionine and hydroxyproline had the least concentrations of 0.1 and 0.04%, respectively.

**TABLE 1:** Amino acid profile of the chicken feathers hydrolysate.

Amino acid	Concentration (%)
Cysteine	0.72
Tryptophan	0.49
Arginine	2.25
Serine	2.03
Aspartic acid	1.01
Glutamic acid	1.65
Glycine	1.93
Threonine	1.11
Alanine	1.11
Tyrosine	0.68
Proline	1.69
Hydroxyproline	0.04
Methionine	0.10
Valine	1.67
Phenylalanine	1.31
Isoleucine	1.05
Leucine	1.71
Histidine	1.29
Lysine	1.41

### Enzyme Characterization

#### Effect of pH on Keratinase Activity and Stability

**FIGURE 3:**
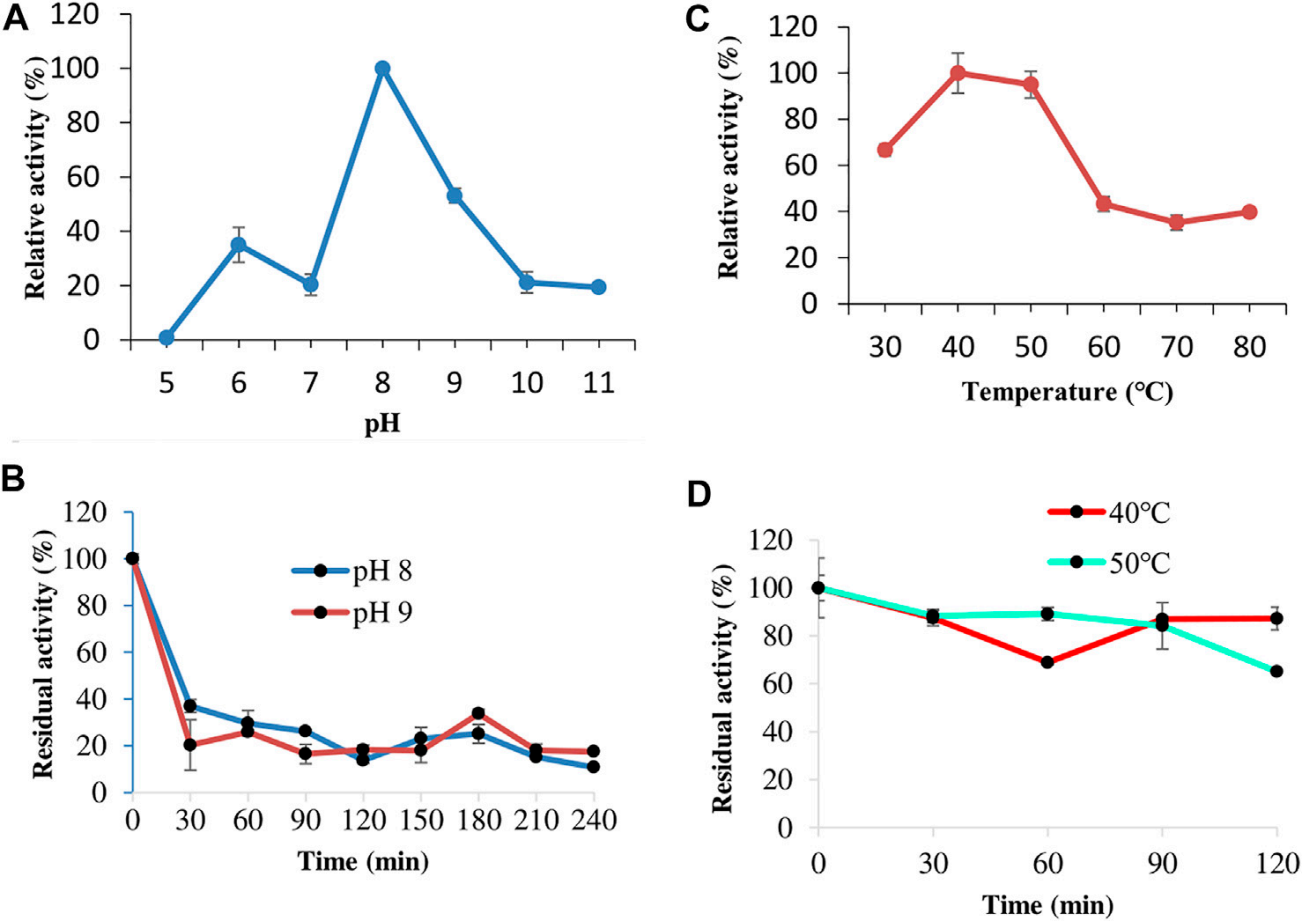
The effect of pH on keratinase activity **(A)** and stability **(B)**. The study of keratinase thermoactivity **(C)** and thermostability **(D)**.

The effect of pH on keratinase activity was studied from pH 5.0–11.0, and the results showed that keratinase from *C*. *aquifrigidense* FANN1 displayed the highest catalytic efficiency at pH 8 (Figure 3A). At pH 9.0, the enzyme activity decreased with 53% relative activity to the optimum. The activity further decreased as the pH tended to strong-alkaline conditions. For the stability study, the keratinase showed a drastic loss of activity after 30 min of preincubation at pH 8.0 and 9.0 (Figure 3B).

#### Effect of Temperature on Keratinase Activity and Stability

The thermoactivity study showed that FANN1 keratinase displayed about 66% relative activity at 30°C (Figure 3C). Further increase in temperature promoted the enzyme catalytic performance, and the maximum enzyme activity was obtained at a range of 40–50°C. Above the optimum temperature, the keratinase activity declined drastically, displaying about 43% relative activity at 60°C. The thermostability evaluation indicated that the keratinolytic protease maintained 87 and 65% of the original activity at 40 and 50°C, respectively, after 120 min of preheating (Figure 3D).

#### Effect of Chemical Agents on Keratinase Stability

The impact of various chemical agents on enzyme catalytic efficiency was evaluated. From the results, *C. aquifrigidense* FANN1 keratinase lost significant enzyme activity in the presence of EDTA and 1,10-phenanthroline, well-known metalloprotease inhibitors (Table 2). However, serine protease inhibitor–PMSF did not alter the catalytic orientation of the protein as it retained 106% of the original activity compared to the control. DTT promoted residual enzyme activity (141 ± 10.38%) in comparison with the control. Likewise, the enzyme was remarkably stable in the presence of the other chemical agents, including hydrogen peroxide, DMSO, acetonitrile, triton X-100, tween-80, and SDS, with residual activity of 98 ± 0.43%, 111 ± 1.73%, 124 ± 0.87%, 104 ± 3.89%, 107 ± 7.79% and 112 ± 0.86%, respectively (Table 2).

**TABLE 2:** Effect of chemical agents on the stability of keratinase from *C. aquifrigidense* FANN1.

Chemical agent	Concentration	Residual activity (%)
Control	-	100 ± 0.43
PMSF	5 mM	106 ± 0
EDTA	5 mM	26 ± 0
1,10-Phenanthroline	5 mM	37 ± 1.73
DTT	5 mM	141 ± 10.38
H_2_O_2_	1% (v/v)	98 ± 0.43
DMSO	1% (v/v)	111 ± 1.73
Acetonitrile	1% (v/v)	124 ± 0.87
Triton X-100	1% (v/v)	104 ± 3.89
Tween-80	1% (v/v)	107 ± 7.79
SDS	5 mM	112 ± 0.87

#### Effect of Metal Ions on Keratinase Stability

Enzyme stability studied in the presence of metal ions (monovalent, divalent, and trivalent) showed that keratinase from *C. aquifrigidense* FANN1 was catalytically enhanced by Fe^3+^ and Na^+^, with residual activity of 120 ± 5.06% and 122 ± 2.95%, respectively (Table 3). The enzyme also showed remarkable stability after the pretreatment with Ca^2+^ (100 ± 10.33%) and Al^3+^ (106 ± 10.33%). Conversely, it showed varying degrees of activity loss in the presence of other metal ions evaluated (Table 3), with the lowest stability recorded against Zn^2+^ (51 ± 8.43%).

**TABLE 3:** Effect of metal ions on the stability of keratinase from *C. aquifrigidense* FANN1.

Metal ion	Concentration (mM)	Residual activity (%)
Control	-	100 ± 1.48
Fe^2+^	5	90 ± 0.42
Co^2+^	5	68 ± 8.22
Fe^3+^	5	120 ± 5.06
K^+^	5	94 ± 6.53
Ca^2+^	5	100 ± 10.33
Mg^2+^	5	88 ± 0.21
Cu^2+^	5	72 ± 1.26
Zn^2+^	5	51 ± 8.43
Na^+^	5	122 ± 2.95
Ba^2+^	5	83 ± 1.69
Al^3+^	5	106 ± 10.33

#### Effect of Laundry Detergent on *C. aquifrigidense* FANN1 Keratinase Stability

**FIGURE 4:**
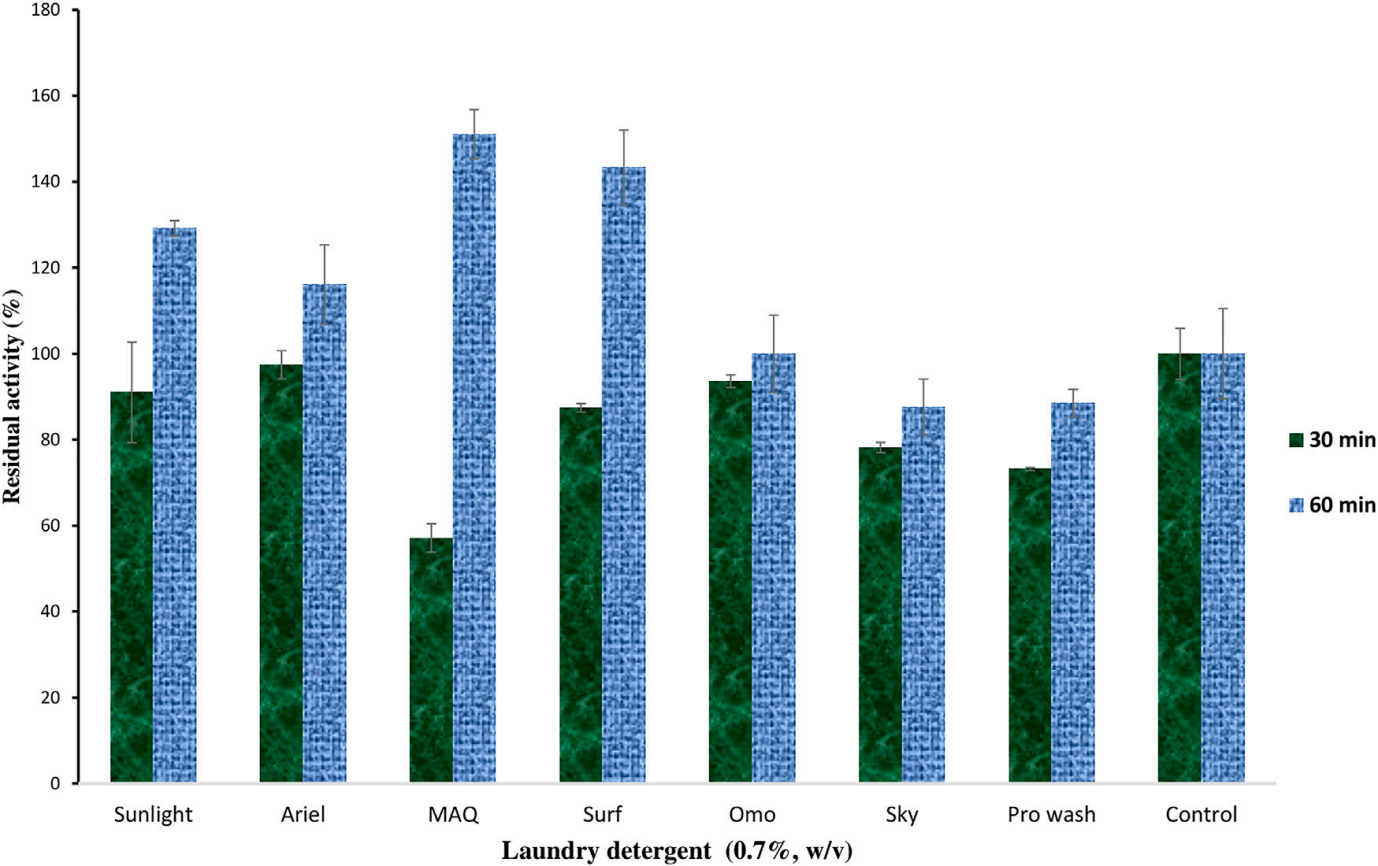
The impact of some selected laundry detergents on the catalytic efficiency of keratinase from *C. aquifrigidense* FANN1.

The impact of selected solid laundry detergents on keratinase stability was investigated. The results showed that keratinolytic protease displayed varying degrees of catalytic stability after 30 min of preincubation with the detergents (Figure 4). The enzyme had <80% residual activity against Sky (78%), Pro wash (73%), and MAQ (57%). However, after 60 min of preincubation, the residual activity was improved across the tested detergents, with significantly catalytic enhancement recorded for Sunlight (129%), Ariel (116%), MAQ (151%), and Surf (143%) compared to the control (Figure 4).

## Discussion

Keratinolytic bacteria are increasingly gaining traction in relevance due to their potential application in a wide range of biotechnological processes. The robust biosynthetic pathways of this group of bacteria conferred them with the dexterity to metabolize recalcitrant keratinous biomass through the extracellular production of keratinolytic enzymes. The versatility and efficiency of keratinase in sustainable development have promoted its market value; hence, exploring various bacterial strains for novel keratinases with improved properties is growing exponentially. The newly isolated *C. aquifrigidense* FANN1 that displayed efficient degradation of intact chicken feathers was used for this study. The construction of optimal fermentation conditions identified weakly acidic conditions as the best pH for maximum keratinase production by *C. aquifrigidense* FANN1. The effect of medium pH on the biosynthetic pathway of microbial secretory proteins has not been fully elucidated. However, it has been established that the interaction between microbial cells and their microenvironment fundamentally regulates various subcompartments of the secretory pathways (Paroutis et al., 2004). These pH-dependent cascade events may include but are not limited to the posttranslational modification and transportation of the synthesized protein. Optimal keratinase production at similar initial pH conditions has also been documented in other studies (Abdel-Fattah et al., 2018; Nnolim et al., 2020c). Contrary to that, several reports have identified neutral to alkaline conditions as the ideal initial pH for an improved keratinase secretion by most keratinolytic bacterial strains (Gurav and Jadhav, 2013b; Bose et al., 2014; Akram et al., 2021). This variation in optimal pH conditions could be attributed to the peculiarities among bacterial isolates.

Inoculum size has been identified as one of the critical factors that influence the extracellular keratinase concentration. Initiation of the fermentation process with proper inoculum size enhances the secretion of keratinolytic protease, promoting the decomposition of keratinous substrates. Conversely, at a higher concentration of starter culture, the extracellular keratinase production was adversely affected. This observation might be due to the overwhelming population of the bacterial culture at early growth stages, leading to the quick depletion of essential growth nutrients (Barman et al., 2017; Akhter et al., 2020). The temperature optimum for keratinase production by *C. aquifrigidense* FANN1 shows a typical mesophilic bacterium with immense potential to develop cost-effective bioprocesses. The majority of keratinolytic *Chryseobacterium* spp. has been reported to show the maximum extracellular keratinase activity at a similar incubation temperature (30°C), including *Chryseobacterium* sp. kr6 (Brandelli and Riffel, 2005), *Chryseobacterium gleum* (Chaudhari et al., 2013), and *Chryseobacterium sediminis* RCM-SSR-7 (Kshetri et al., 2019). However, other moderately thermophilic bacteria that maximally secreted keratinase between 50 and 65°C have been documented (Bihari et al., 2010; Gurav and Jadhav, 2013b; Cavello et al., 2018); and they were isolated from either temperate or hot spring region. The optimal chicken feather concentration for the maximum keratinase production agrees with similar studies in the literature (Bose et al., 2014; Akram et al., 2021; Prajapati et al., 2021). Keratinase is generally produced by induction in the presence of keratinous materials. Hence, keratinase titre would be expected to show linear function with the concentration of the substrates. Contrarily, keratinase secretion tend to drop with increasing concentration of keratinous biomass, and this phenomenon has been attributed to some factors, including high viscosity and poor medium aeration (Bose et al., 2014), and downregulation of keratinase–encoding gene due to high concentration of bioaccessible products (Nnolim and Nwodo, 2020).

The kinetics of keratinase production by *C. aquifrigidense* FANN1 showed maximum keratinase concentration between 72 and 96 h of the incubation period, during which the viable cell concentrations decreased significantly. The increased keratinase titre may be due to the liberation of an intracellularly trapped enzyme by virtue of cell membrane disruption. Similarly, *C. gleum* (Chaudhari et al., 2013) and *C. sediminis* RCM-SSR-7 (Kshetri et al., 2019) exhibited the highest extracellular keratinase activity at 72 and 84 h, respectively. Contrariwise, optimal keratinase production by *Chryseobacterium indologenes* A22 (Bach et al., 2015), *Chryseobacterium* sp. P1-3 (Hong et al., 2015) and *Chryseobacterium* sp. RBT (Gurav et al., 2016) was recorded at 40, 48, and 48 h, respectively. The total protein quantified over the time course study indicated that the soluble proteins emanated from either keratin degradation or microbial metabolism increasingly accumulated in the medium as the fermentation progressed. In the same vein, the fermentation medium’s pH drifts from weakly acidic to neutral-alkaline condition indicates the constant generation of ammonia from deamination of soluble proteins in the fermentation medium (He et al., 2018). The change in pH is a peculiarity of promising proteolytic strains growing on keratinous materials (Nnolim et al., 2020b). Furthermore, thiol group detection in the keratin fermentation medium suggests hydrolysis of cysteine disulfide bonds, which increases the susceptibility of the biopolymer to proteolytic attack (Gurav et al., 2016; He et al., 2018). An elevated concentration of the thiol group obtained in the present study after 48 h signifies the efficiency of the keratinolytic and/or sulfitolytic systems of *C. aquifrigidense* FANN1.

The hydrolysate analysis provided helpful information about the amino acid liberation during the cultivation of *C. aquifrigidense* FANN1 on keratinous chicken feathers. The variation between the concentration of the total free amino acids and the hydrolysate’s protein value could result from the presence of short peptides and other non-protein nitrogenous compounds. The amino acid profile of the protein hydrolysate underpins its significance as an alternative low-cost animal feed supplement to the expensive soybean meal, which has been fundamentally used as a feed-grade dietary protein source. Similarly, protein hydrolysate from *C. sediminis* RCM-SSR-7 assisted feather degradation showed higher concentrations of arginine and serine, followed by leucine, aspartic acid, and phenylalanine (Kshetri et al., 2019). Furthermore, keratinolytic *Chryseobacterium* sp. RBT-assisted feather conversion showed medium amino acid accumulation as a function of time, and this observation was attributed to further hydrolysis of peptides into free amino acids (Gurav et al., 2016).

The investigation of temperature and pH optima for *C. aquifrigidense* FANN1 keratinase showed that the enzyme’s maximal catalytic efficiency conditions were consonant with other *Chryseobacterium* spp. keratinases already characterized. Keratinases from *Chryseobacterium* sp. kr6 (Riffel et al., 2007), *C. indologenes* (Bach et al., 2011), *Chryseobacterium* L99 sp. nov. (Lv et al., 2010), and *Chryseobacterium* sp. P1-3 (Hong et al., 2015) exhibited optimal catalysis at pH and temperature of 8.5 and 50°C, 7.5 and 45–55°C, 8.0 and 40°C, and 8.0 and 30°C, respectively. The thermostability study showed that the enzyme could be used at moderately high-temperature conditions over a given time with significant biocatalytic efficiency. However, the enzyme demonstrated low pH stability post-incubation at buffers of pH 8.0 and 9.0, and the loss of stability may be attributed to the change in catalytic orientation of the enzyme structure through the protonation pattern of the essential residues and side acids involved in the coordination of catalysis (Nnolim and Nwodo, 2020). Metagenomic studies have shed more light on *Chryseobacterium* species’ crucial roles in the biodegradation of recalcitrant keratinous substrates (Kang et al., 2018; Kang et al., 2021). Therefore, the keratinolytic protease would enjoy immense industrial and biotechnological relevance if the stability is improved using a protein engineering approach (Fang et al., 2016; Nnolim et al., 2020a).

The sensitivity of the keratinolytic protease to the protease inhibitors suggests that it belongs to the metalloprotease class. Similarly, few characterized keratinases of *Chryseobacterium* origin have been identified as metallo-keratinases (Riffel et al., 2007; Bach et al., 2011; Pereira et al., 2014). Reducing agents have been reported to interfere with the disulfide bonds that ensure proteases’ structural integrity, while organic solvents disrupt catalytic conformation by disrupting the hydrophobic interactions among the nonpolar side chains (Fang et al., 2013). The remarkable residual activity of the enzyme post-treatment with a reducing agent, oxidizing agent, organic solvents, and surfactants highlights the enzyme’s application potential as an industrial bio-additive, especially where these chemical agents are crucial to the process implementation (Fakhfakh-Zouari et al., 2010; Rai and Mukherjee, 2011). Metallo-keratinase from *Chryseobacterium* sp. kr6 showed a similar stability pattern in the presence of triton X-100 and tween-80 (Riffel et al., 2003). On the contrary, *C. indologenes* A22 keratinase was drastically affected by triton X-100 and completely lost activity after DTT pretreatment even at lower concentrations (Bach et al., 2011).

The keratinolytic protease displayed residual activity that varied among the respective metal ions tested, with the most stimulatory effect was obtained for Fe3+ and Na+, while the repressive effect was recorded against Zn^2+^. Metallo-proteases mostly have inherent Zn^2+^ that modulates the protein’s catalytic property (Thys and Brandelli, 2006; Riffel et al., 2007; Wang et al., 2008). Therefore, an extra Zn^2+^ concentration could change the catalytic orientation of the protein by binding to other sites where it is not needed and causing structural destabilization through direct association with the residues or allosteric regulation. This submission may explain why zinc (II) ion caused more inhibition of the catalytic performance of the enzyme than other heavy and transition metal ions used. A comparable pattern of enzyme activity inhibition at a similar concentration of the metal ions has been reported for metallo-keratinases in other studies (Thys and Brandelli, 2006; Tork et al., 2013; Zhang et al., 2016).

Keratinolytic peptidase inclusion in the detergent formulation has significantly improved the washing performance of detergents than other classical proteases as it promotes the destaining of both soluble and insoluble proteinaceous stains while withstanding the influences of harsh washing conditions and detergent ingredients (Paul et al., 2014). The *C. aquifrigidense* FANN1 keratinase generally exhibited a high degree of tolerance to the various detergents tested. The endogenous protease of detergents shows inconsistent stability due to variations in ingredients and concentrations among different detergent brands (Reddy et al., 2017). The residual activity displayed by FANN1 keratinase after 60 min of pretreatment is higher compared to *Arthrobacter* sp. KFS-1 keratinase in a similar detergent panel (Nnolim et al., 2020d). The detergent tolerability of *C. aquifrigidense* FANN1 keratinase is worthy of further investigation for potential applicability in the detergent formulation.

In conclusion, keratinolytic *C. aquifrigidense* FANN1 isolated from a local poultry dumpsite was used to produce keratinase in a medium constituted with chicken feathers as the sole carbon and nitrogen source. FANN1 showed maximal keratinase titre after 72 h at optimized physicochemical conditions. The feather hydrolysate analysis showed that free amino acids were liberated from feather keratin, which underpins the isolate’s prowess in the valorization of keratin-rich agroindustrial residues. The enzyme was optimally active at pH 8.0 and 40–50°C. The sensitivity of the enzyme to protease inhibitors suggested a metallo-keratinase, with a remarkable tolerance of surfactants, organic solvents, reducing and oxidizing agents. Also, FANN1 keratinolytic protease showed excellent residual activity post-incubation with various commercially available laundry detergents, which indicates its significance as a potential bio-additive for detergent formulation. Thus, the keratinolytic propensity of *C. aquifrigidense* FANN1 is promising for bio-innovative developments. Prospectively, whole-genome sequencing and mining of keratinolytic determinants would be crucial to exploiting the bacterium further. Therefore, cloning, heterologous expression, and molecular optimization of the keratinolytic determinants are promising to enhance the suitability and versatility of the keratinolytic protease in a vast array of industrial and biotechnological developments.

## Data Availability

The original contributions presented in the study are included in the article/Supplementary Material, further inquiries can be directed to the corresponding author.
